# Validity of the γ-Ray Evaluation with iodoamphetamine for Cerebral Blood Flow Assessment (REICA) method for quantification of cerebral blood flow including acetazolamide challenge test

**DOI:** 10.1007/s12149-021-01700-w

**Published:** 2022-01-01

**Authors:** Yoshiaki Miyazaki, Masashi Kameyama, Akira Nakamizo, Tomoyuki Noguchi, Nobuyuki Tabata

**Affiliations:** 1 Department of Radiology, National Hospital Organization Kyushu Medical Center, 1-8-1, Jigyohama, Chuo-Ku, Fukuoka-City, Fukuoka 810-8563 Japan; 2 Present Address: Department of Radiological Technology, Radiological Diagnosis, National Cancer Center, 5-1-1, Tsukiji, Chuo-Ku, Tokyo, 104-0045 Japan; 3 Department of Diagnostic Radiology, Tokyo Metropolitan Geriatric Hospital and Institute of Gerontology, 35-2 Sakae-Cho, Itabashi-Ku, Tokyo, 173-0015 Japan; 4 Department of Neurosurgery, Clinical Research Institute, National Hospital Organization Kyushu Medical Center, 1-8-1, Jigyohama, Chuo-Ku, Fukuoka-City, Fukuoka 810-8563 Japan

**Keywords:** Cerebral blood flow, Cerebrovascular reactivity, ^123^I-IMP, Single-photon emission computed tomography

## Abstract

**Objective:**

The γ-Ray Evaluation with iodoamphetamine for Cerebral Blood Flow Assessment (REICA) is a new method for quantifying cerebral blood flow (CBF) using single-photon emission computed tomography (SPECT) and [^123^I]*N*-isopropyl-*p*-iodoamphetamine (^123^I-IMP). The present study aimed to validate the REICA method using data including acetazolamide challenge test.

**Methods:**

The REICA and Graph-Plot (GP) methods were used to calculate mean CBF (mCBF) for 92 acquisitions (rest: 57, stress: 35) and cerebrovascular reactivity (CVR) in 33 patients. To obtain stress data, 15 mg/kg of acetazolamide was injected intravenously 10 min before the administration of ^123^I-IMP, and blood samples were collected under the same conditions as rest data. The reference standard was the Autoradiograph (ARG) method using arterial blood sampling, and the accuracy of the REICA method was analyzed by comparing it with each method.

**Results:**

For mCBF, the correlation coefficients (*r*) were 0.792 for the REICA method and 0.636 for the GP method. For CVR, r values were 0.660 for the REICA method and 0.578 for the GP method. In both acquisitions, the REICA method had a stronger correlation with the ARG method than the GP method. For mCBF, there was a significant difference in the correlation coefficient between the two correlation coefficients (*p* < 0.01).

**Conclusions:**

The REICA method was more accurate than the GP method in quantifying CBF and closer to the ARG method. The REICA method, which is a noninvasive method of cerebral blood flow quantification using ^123^I-IMP, has great medical usefulness.

## Introduction

Single-photon emission computed tomography (SPECT), which evaluates ischemic cerebrovascular disease [1] and diagnoses dementia [2], can aid in determining treatment strategies as well as therapeutic and surgical effects through analyses of cerebral blood flow (CBF) [3]. Furthermore, the combination of “stress” data obtained during CBF loading with acetazolamide (ACZ) and “rest” data during resting CBF can be used to calculate cerebrovascular reactivity (CVR), which can in turn be used to predict the patients at high risk for perioperative hyperperfusion [4–9].

There are different types of cerebral blood flow SPECT tracers, such as ^99m^Tc-hexamethylpropyleneamine oxime (^99m^Tc-HMPAO) and ^99m^Tc-ethyl cysteinate dimer (^99m^Tc-ECD). [^123^I]N-isopropyl-p-iodoamphetamine (^123^I-IMP) is an ideal tracer in that it does not underestimate regional cerebral blood flow (rCBF) at high blood flow values [10].

In the quantification of cerebral blood flow by SPECT, the Autoradiograph (ARG) method [11] is a simplified version of the Table Look Up method [12] based on a one-tissue, two-compartment model. Currently, the ARG method, which requires arterial blood sampling, is the standard method for CBF quantification using ^123^I-IMP.

Graph-Plot (GP) method [13], another method of CBF quantification that does not use arterial blood sampling has some serious methodological flaws. It is not appropriate to use pulmonary artery measurements as the input function [14].

Recently, a new CBF quantification method using SPECT and ^123^I-IMP has been proposed [14]. The γ-Ray Evaluation with iodoamphetamine for Cerebral blood flow Assessment (REICA) method does not require invasive arterial blood sampling, nor does it require the use of a regression formula. Moreover, the method removes bias due to the variable pulmonary tracer retention by accounting for it within the equation. However, there are only two reports on the REICA method [14, 15], and they only validated the REICA method on rest data using a single γ-camera, and no reports of stress data obtained under ACZ loading have been published yet.

Therefore, in the present study, we aimed to validate the REICA method.

## Materials and methods

### Participants

A total of 59 patients (22 males and 37 females; mean age 59.7 ± 17.5) who underwent simultaneous GP and ARG procedures between August 2018 and November 2019 were included in the study (Table 1). Rest/stress examinations of the same patient were performed at intervals of 7 days or less. Diseases among the included participants were Moyamoya disease (18 patients), middle cerebral artery stenosis or occlusion (20 patients), carotid artery stenosis or occlusion (16 patients), and other vascular disorders (5 patients).

**Table 1:** Patient characteristics

	Number of patients	Rest and stress	Rest	Stress	Average age (year)
Male	22	11	11	0	69.5 ± 13.9
Female	37	22	13	2	54.0 ± 16.8
Total	59	33	24	2	59.7 ± 17.5

± denotes standard deviation

For the clinical images in this study, care was taken to protect personal information to prevent the identification of participants. This study was conducted in accordance with the Declaration of Helsinki 1975, as revised in 2000. The Ethics Review Committee of National Hospital Organization Kyushu Medical Center approved the utilization of data (Approval no. 19C203) and waived the need for patient consent for the utilization of existing data (the retrospective study).

### Data acquisition

**Fig. 1:**
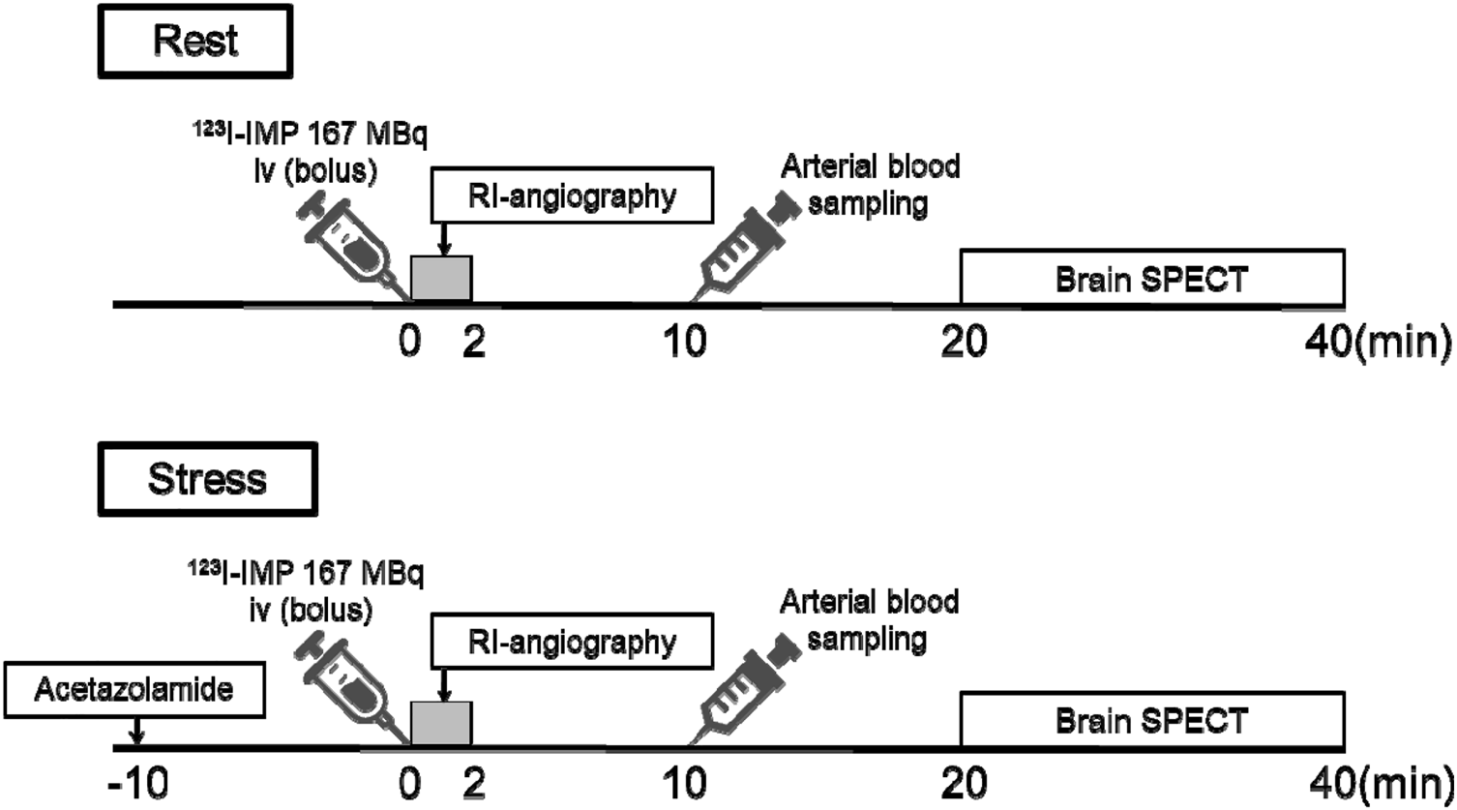
Protocol details (rest and stress)

The dynamic planar protocol (Fig. 1) was used as previously reported [14]. Dynamic frontal planar scans (2 s/frame, 60 frames, 3.9 mm/pixel) were acquired using a γ-camera (Siemens E.CAM, Siemens Medical Solutions, Erlangen, Germany) equipped with a low-energy high-resolution collimator from intravenous administration of a 167 MBq ^123^I-IMP. The rest and stress data were acquired using the same γ-camera. Arterial blood samples were measured for γ-ray radiation counts using a well-type scintillation measurement device (NDW-351F; Hitachi Aloka Medical, Ltd.). The arterial blood samples were taken 10 min after the ^123^I-IMP administration, and the input function values were obtained from the average of four measurements of radioactivity concentration per 1.0 g. To obtain stress data, 15 mg/kg of ACZ was injected intravenously 10 min before the administration of ^123^I-IMP, and blood samples were collected under the same conditions as rest data. The image reconstruction method is filtered back projection, the reconstruction filter is ramp, and the preprocessing filter is Butterworth (cutoff 0.35 cycle/cm; order 8). Chang’s method was adopted for attenuation correction, and the attenuation coefficient was set to 0.07/cm. Scattering correction was not performed.

### Data analysis

The REICA method was used for data analysis in this study. Regions of interest (ROIs) were selected for the pulmonary artery trunk, brain, and lungs. The time-activity curves (TACs) calculated by the ROI was smoothed with a low-pass filter to reduce statistical noise. After that, a time zero adjustment was performed when sharp increases in activity were observed [13, 16]. In this study, $\left( {\frac{{\mathop \smallint \nolimits_{0}^{t} C_{{\text{r}}} \left( \tau \right){\text{d}}\tau }}{l\left( t \right)},\;\frac{{C_{{\text{b}}} \left( t \right)}}{\lambda l\left( t \right)}} \right)$ was plotted, and the slope of the linear section was determined. *C*_r_(*t*) denotes tracer activity in the pulmonary artery, *l*(*t*) denotes activity in the lungs, *C*_b_(*t*) denotes activity in the brain, and *λ* denotes lipophilic fraction of ^123^I-IMP [14, 15].

The ARG method was performed using the built-in software (Syngo MI Applications®, Siemens Medical Solutions, Erlangen, Germany). The volume of distribution (Vd) value was set to 40. For the quantitative SPECT images obtained by the ARG method, ROIs were set on the normal side of the brain parenchyma nuclei in the basal ganglia slice, and mean CBF (mCBF) by the ARG method was obtained.

The GP method was analyzed using dedicated software Hayabusa (AZE VirtualPlace Hayabusa®, AZE, Tokyo, Japan). For the analysis, ROIs were set in the pulmonary artery and basal ganglia, respectively. For each ROI, the standardized F (SFR, F = Cerebral blood flow index) was obtained using the TACs of the pulmonary artery as the input function and the TACs of the brain as the output function. The mCBF was calculated from SFR using a conversion formula. The conversion equation for the GP method is as follows [17]:

1 $$ {\text{mCBF }} = { 1}.{\text{87 SFR }} + { 21}.{2}{\text{.}} $$

The CVR for stress data obtained after injection of ACZ is calculated using the following equation [6]:

2 $$ {\text{CVR}}(\% ) = \frac{{{\text{mCBF}}_{{{\text{stress}}}} - {\text{mCBF}}_{{{\text{rest}}}} }}{{{\text{mCBF}}_{{{\text{rest}}}} }} \times 100. $$

The mCBF value acquired using the ARG method was used as the reference standard.

### Statistical analysis

The data, including significance levels, were analyzed using Excel® 2016 (Microsoft Corporation, Redmond, WA, USA).

## Results

### mCBF

**Fig. 2:**
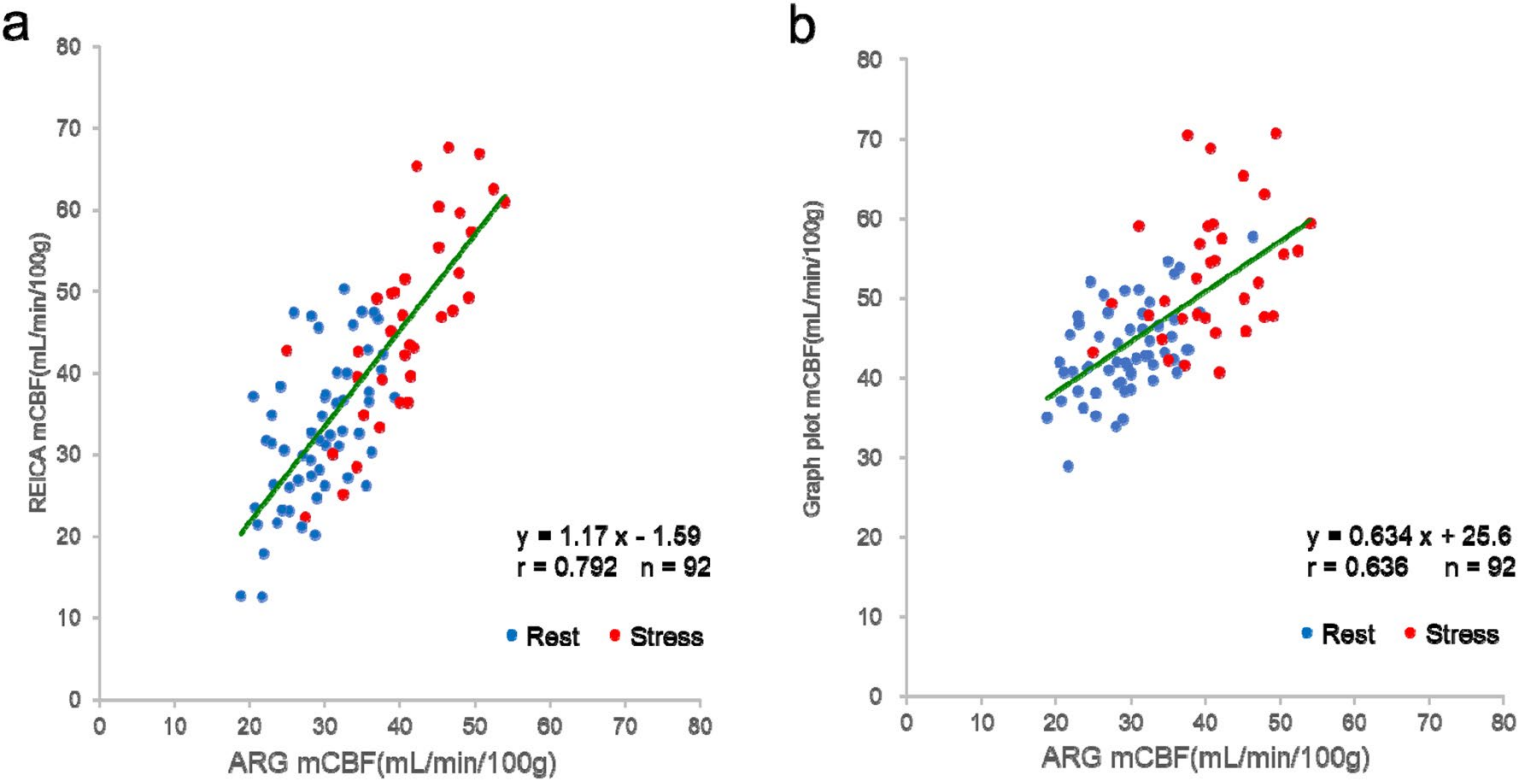
Comparison of mean cerebral blood flow (mCBF) using the autoradiograph (ARG) method for 92 acquisitions. **a** Comparison between the γ-Ray Evaluation with Iodoamphetamine for Cerebral Blood Flow Assessment (REICA) and ARG methods. **b** Comparison between the graph-plot (GP) and ARG methods

Figure 2 shows the relationship between mCBF measured using the REICA and ARG method and mCBF measured using the GP and ARG method for 92 acquisition (rest: 57, stress: 35). The linear regression and correlation coefficients (*r*) were as follows: REICA and ARG method, *y* = 1.17 *x−*1.59, *r* = 0.792, *p* = 5.96 × 10^–21^; GP and ARG method, *y* = 0.634 *x* + 25.6, *r* = 0.636, *p* = 1.01 × 10^–11^. Furthermore, the difference between the two correlation coefficients was significant at *p* = 7.30 × 10^–3^, indicating that the REICA method had a stronger correlation with the ARG method than the GP method. The slope of the linear regression for the REICA method was closer to 1.0 and the intercept to 0.0 than those of the linear regression for the GP method.

### CVR

**Fig. 3:**
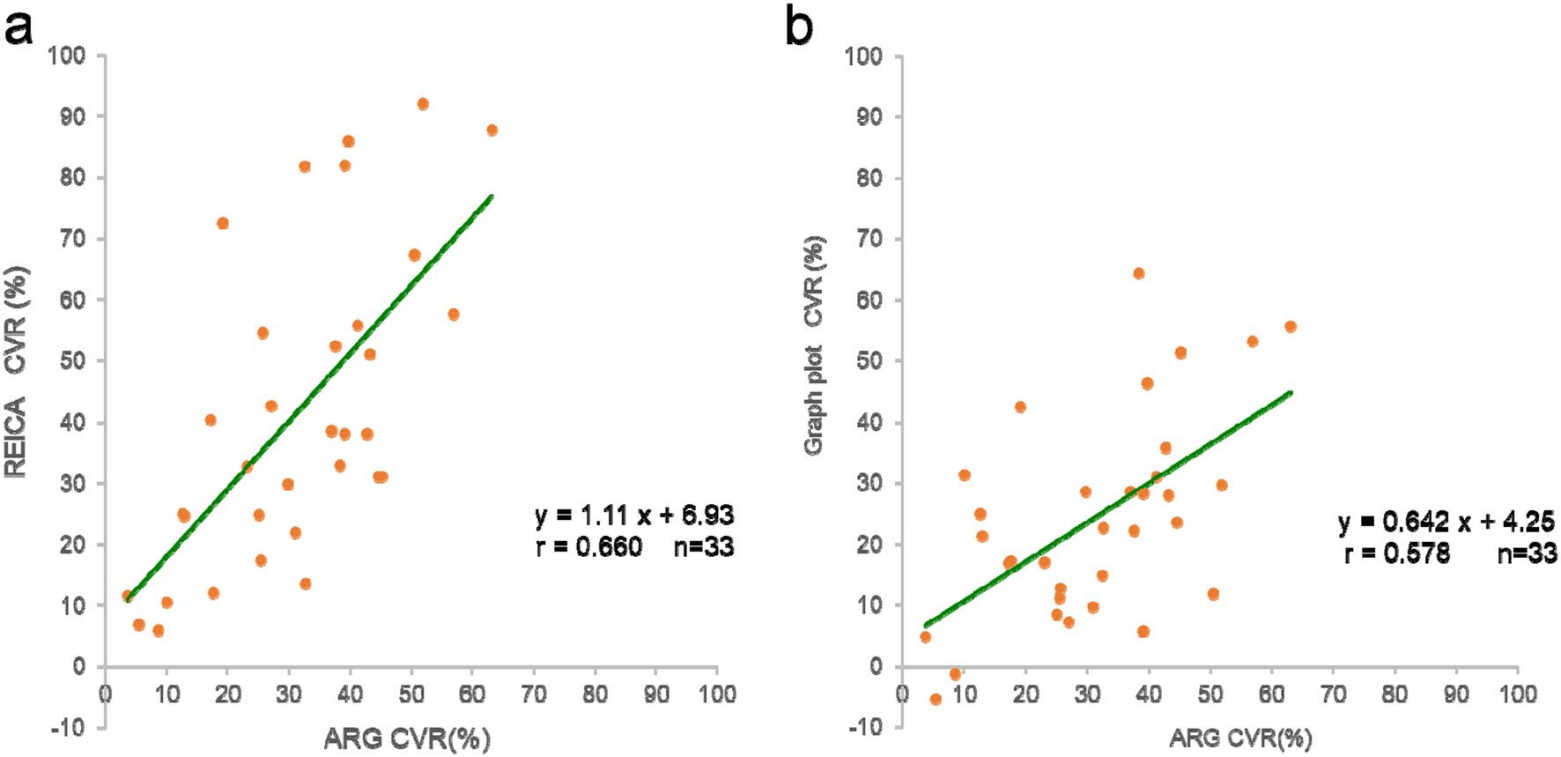
Comparison of cerebrovascular reactivity (CVR) using the autoradiograph (ARG) method in 33 patients. **a** Comparison between the γ-Ray Evaluation with Iodoamphetamine for Cerebral Blood Flow Assessment (REICA) and ARG methods. **b** Comparison between the graph-plot (GP) and ARG methods

Figure 3 shows the results of the CVRs of REICA and ARG, of GP and ARG for the data of 33 patients. The respective linear regression and correlation coefficients were as follows: REICA and ARG method, *y* = 1.11 *x* + 6.93, *r* = 0.660, *p* = 2.96 × 10^–5^; GP and ARG method, *y* = 0.642 *x* + 4.25, *r* = 0.578, *p* = 4.28 × 10^–4^. Compared with the GP method, the REICA method yielded positive CVR values, and the slope of the linear regression equation was close to 1.0.

## Discussion

The present study is the first to verify the REICA method for calculating CBF and CVR using data including acetazolamide challenge test. Our findings indicated that, especially for mCBF, there was a significant difference between the two correlation coefficients (*p* < 0.01), proving the high accuracy of the REICA method. Adding this study using Siemens SPECT machine to the previous studies using GE machine, we hope that REICA method can be generalized. In addition, during acetazolamide administration, the blood flow tracer is rapidly transferred to the brain. The CBV is about 3–5% [18], and the effect of the change in CBV on the mCBF measurement measured by the REICA and GP methods would be minimal.

The linear regression equation for mCBF determined using the GP method exhibited a low slope (0.634, REICA: 1.17) and high intercept (25.6, REICA: − 1.59), suggesting that mCBF was overestimated when low. The conversion formula used in the GP method when calculating mCBF was mainly constructed from the rest dataset [13], which may have caused a discrepancy in the quantitative values in high blood flow, and the mCBF values in the stress data are likely to be underestimated. In other words, the use of a conversion formula that is not appropriate for each condition increases the possibility of a mCBF error.

Furthermore, in the calculation of CVR using Eq. (1), overestimation of mCBF_rest_ and underestimation of mCBF_stress_ can result in further underestimation of CVR, which can lead to the incorrect judgment that there is a risk of overperfusion. Previous studies have reported that CVR obtained based on SPECT analysis of CBF using ACZ loading can help to identify patients at high risk of recurrent stroke [5, 6], and to predict hyperperfusion in patients undergoing carotid endarterectomy [7, 8] and carotid artery stenting [9, 10]. These studies highlight the importance of this index in the perioperative complication cycle.

REICA method may be susceptible to body weight and the distance between the camera and body. The input function of a heavier person may be underestimated due to the attenuation of the γ-Ray, which would lead to the overestimation of CBF. A parallel hole collimator would not affect the counts, however, the longer distance between the camera and the body would make the image blur, which may lead to underestimation of the counts. The same phenomenon would be considered to be occurred in the GP method and the Gjedde–Patlak–Matsuda method. Further studies would be needed to confirm this speculation.

The present study has a limitation. The number of patients, especially who underwent both rest and stress tests were not large, because the participants were recruited from a single hospital.

## Conclusion

The present validation was performed by adding REST data to the stress data, which has not been reported so far in the REICA method. It was found that the quantification of CBF by the REICA method was more accurate than the GP method, which was closer to the ARG method. Thus, our findings highlight the medical potential of REICA method for noninvasive quantification of CBF using ^123^I-IMP.
